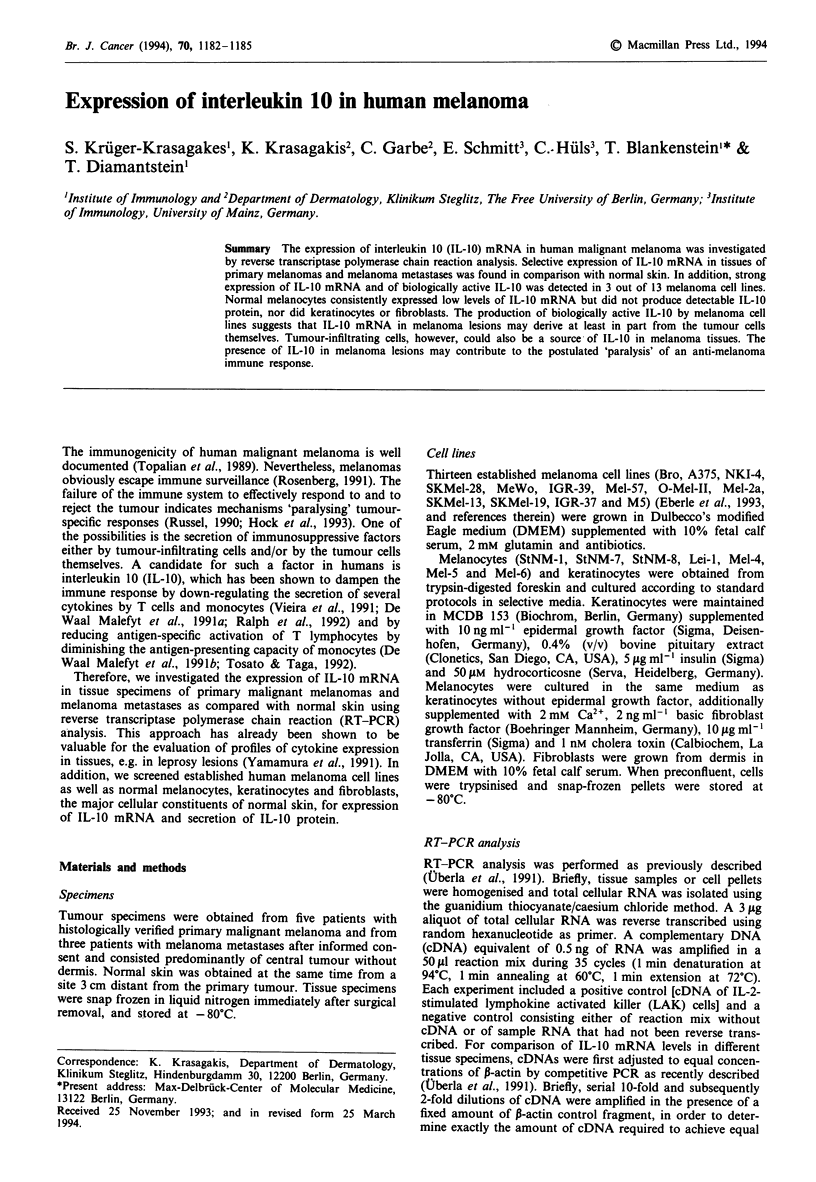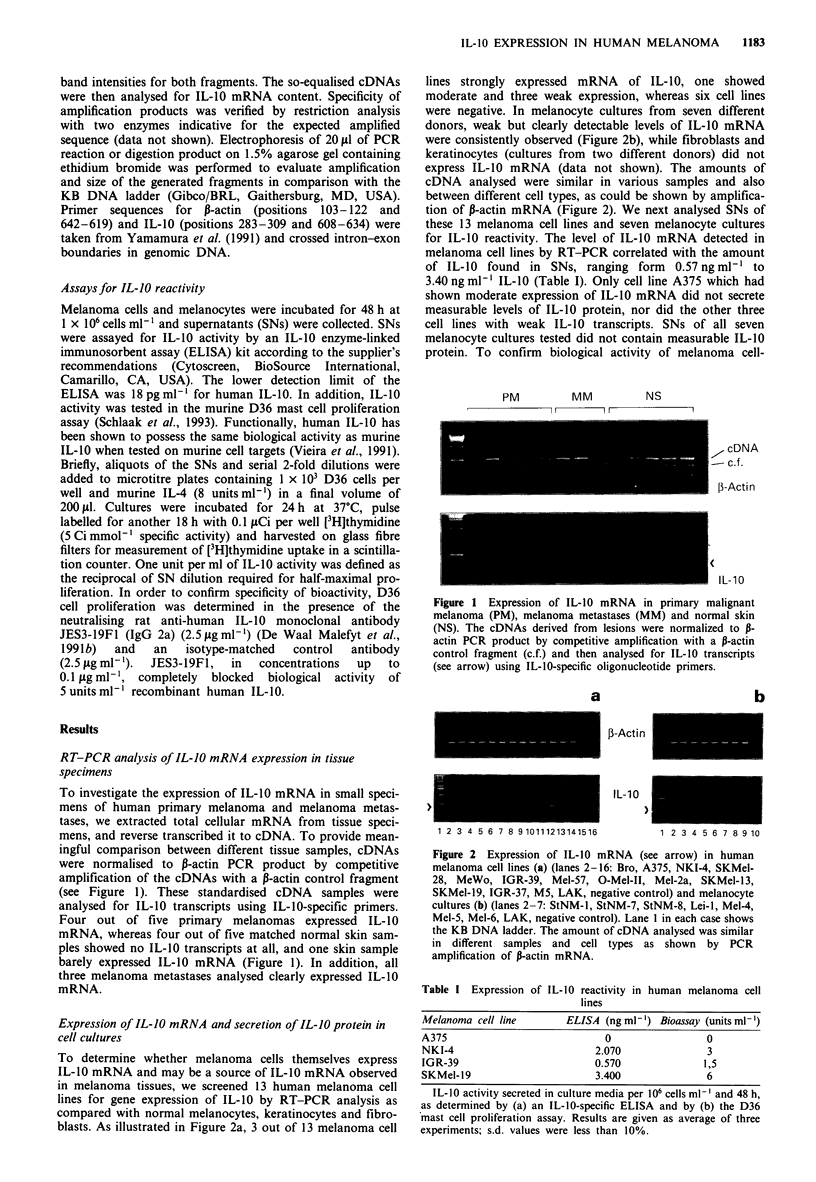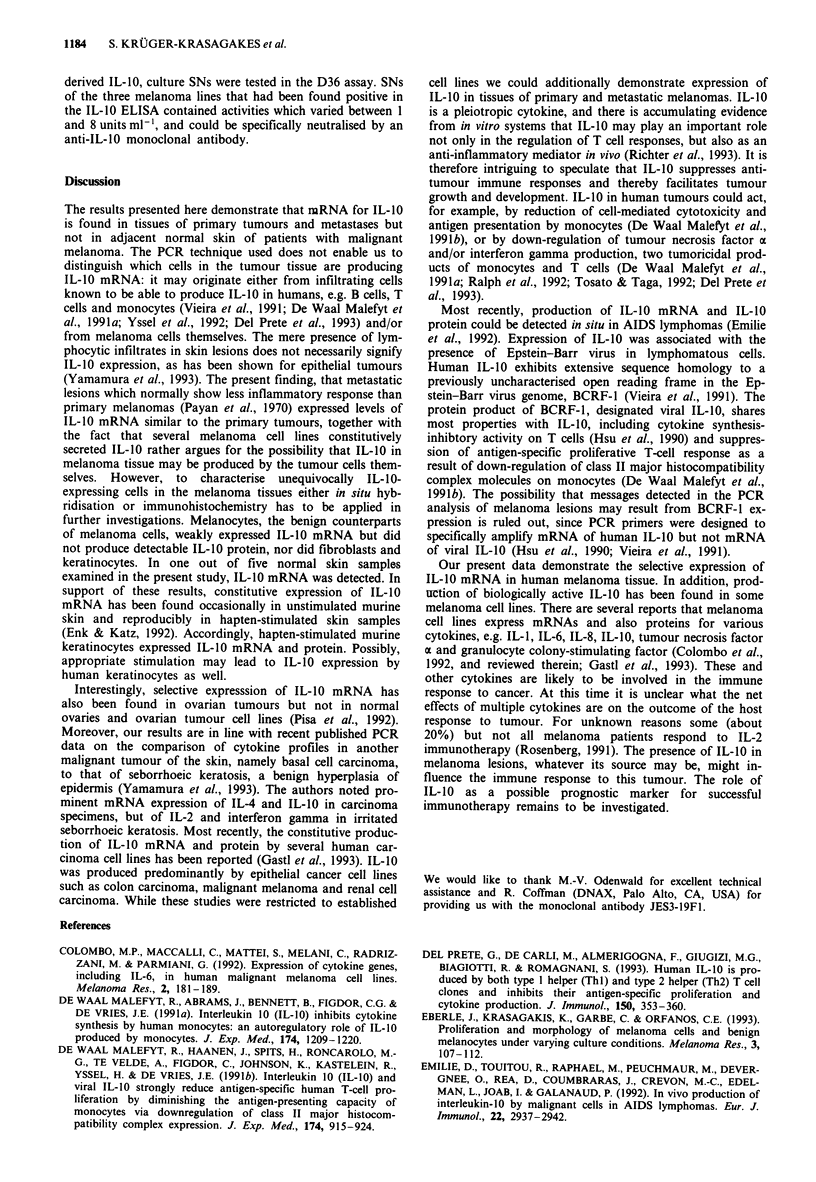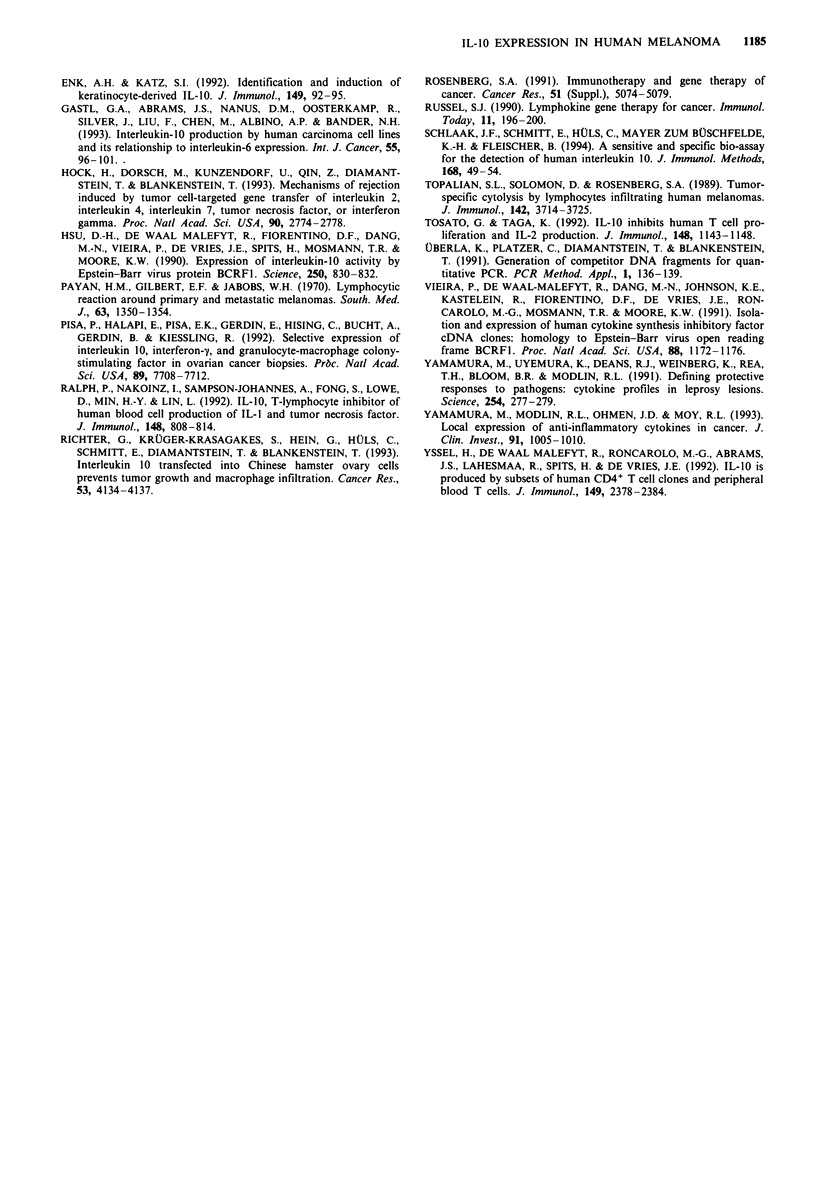# Expression of interleukin 10 in human melanoma.

**DOI:** 10.1038/bjc.1994.469

**Published:** 1994-12

**Authors:** S. Krüger-Krasagakes, K. Krasagakis, C. Garbe, E. Schmitt, C. Hüls, T. Blankenstein, T. Diamantstein

**Affiliations:** Institute of Immunology, Free University of Berlin, Germany.

## Abstract

**Images:**


Br. J. Cancer (1994), 70, 1182–1185 © Macmillan Press Ltd., 1994

### Expression of interleukin 10 in human melanoma

S. Krüger-Krasagakes[1], K. Krasagakis[2], C. Garbe[2], E. Schmitt[3], C.-Hüls[3], T. Blankenstein[1]* & T. Diamantstein[1]

[1]*Institute of Immunology and* [2]*Department of Dermatology, Klinikum Steglitz, The Free University of Berlin, Germany;* [3]*Institute of Immunology, University of Mainz, Germany.*

**Summary** The expression of interleukin 10 (IL-10) mRNA in human malignant melanoma was investigated by reverse transcriptase polymerase chain reaction analysis. Selective expression of IL-10 mRNA in tissues of primary melanomas and melanoma metastases was found in comparison with normal skin. In addition, strong expression of IL-10 mRNA and of biologically active IL-10 was detected in 3 out of 13 melanoma cell lines. Normal melanocytes consistently expressed low levels of IL-10 mRNA but did not produce detectable IL-10 protein, nor did keratinocytes or fibroblasts. The production of biologically active IL-10 by melanoma cell lines suggests that IL-10 mRNA in melanoma lesions may derive at least in part from the tumour cells themselves. Tumour-infiltrating cells, however, could also be a source of IL-10 in melanoma tissues. The presence of IL-10 in melanoma lesions may contribute to the postulated 'paralysis' of an anti-melanoma immune response.

The immunogenicity of human malignant melanoma is well documented (Topalian *et al.*, 1989). Nevertheless, melanomas obviously escape immune surveillance (Rosenberg, 1991). The failure of the immune system to effectively respond to and to reject the tumour indicates mechanisms 'paralysing' tumour-specific responses (Russel, 1990; Hock *et al.*, 1993). One of the possibilities is the secretion of immunosuppressive factors either by tumour-infiltrating cells and/or by the tumour cells themselves. A candidate for such a factor in humans is interleukin 10 (IL-10), which has been shown to dampen the immune response by down-regulating the secretion of several cytokines by T cells and monocytes (Vieira *et al.*, 1991; De Waal Malefyt *et al.*, 1991*a*; Ralph *et al.*, 1992) and by reducing antigen-specific activation of T lymphocytes by diminishing the antigen-presenting capacity of monocytes (De Waal Malefyt *et al.*, 1991*b*; Tosato & Taga, 1992).

Therefore, we investigated the expression of IL-10 mRNA in tissue specimens of primary malignant melanomas and melanoma metastases as compared with normal skin using reverse transcriptase polymerase chain reaction (RT–PCR) analysis. This approach has already been shown to be valuable for the evaluation of profiles of cytokine expression in tissues, e.g. in leprosy lesions (Yamamura *et al.*, 1991). In addition, we screened established human melanoma cell lines as well as normal melanocytes, keratinocytes and fibroblasts, the major cellular constituents of normal skin, for expression of IL-10 mRNA and secretion of IL-10 protein.

### Materials and methods

### *Specimens*

Tumour specimens were obtained from five patients with histologically verified primary malignant melanoma and from three patients with melanoma metastases after informed consent and consisted predominantly of central tumour without dermis. Normal skin was obtained at the same time from a site 3 cm distant from the primary tumour. Tissue specimens were snap frozen in liquid nitrogen immediately after surgical removal, and stored at −80°C.

Correspondence: K. Krasagakis, Department of Dermatology, Klinikum Steglitz, Hindenburgdamm 30, 12200 Berlin, Germany.
*Present address: Max-Delbrück-Center of Molecular Medicine, 13122 Berlin, Germany.
Received 25 November 1993; and in revised form 25 March 1994.

### *Cell lines*

Thirteen established melanoma cell lines (Bro, A375, NKI-4, SKMel-28, MeWo, IGR-39, Mel-57, O-Mel-II, Mel-2a, SKMel-13, SKMel-19, IGR-37 and M5) (Eberle *et al.*, 1993, and references therein) were grown in Dulbecco's modified Eagle medium (DMEM) supplemented with 10% fetal calf serum, 2 mM glutamin and antibiotics.

Melanocytes (StNM-1, StNM-7, StNM-8, Lei-1, Mel-4, Mel-5 and Mel-6) and keratinocytes were obtained from trypsin-digested foreskin and cultured according to standard protocols in selective media. Keratinocytes were maintained in MCDB 153 (Biochrom, Berlin, Germany) supplemented with 10 ng $ml^{-1}$ epidermal growth factor (Sigma, Deisenhofen, Germany), 0.4% (v/v) bovine pituitary extract (Clonetics, San Diego, CA, USA), 5 $\mu$g $ml^{-1}$ insulin (Sigma) and 50 $\mu$M hydrocorticosne (Serva, Heidelberg, Germany). Melanocytes were cultured in the same medium as keratinocytes without epidermal growth factor, additionally supplemented with 2 mM $Ca^{2+}$, 2 ng $ml^{-1}$ basic fibroblast growth factor (Boehringer Mannheim, Germany), 10 $\mu$g $ml^{-1}$ transferrin (Sigma) and 1 nM cholera toxin (Calbiochem, La Jolla, CA, USA). Fibroblasts were grown from dermis in DMEM with 10% fetal calf serum. When preconfluent, cells were trypsinised and snap-frozen pellets were stored at −80°C.

### *RT–PCR analysis*

RT–PCR analysis was performed as previously described (Überla *et al.*, 1991). Briefly, tissue samples or cell pellets were homogenised and total cellular RNA was isolated using the guanidium thiocyanate/caesium chloride method. A 3 $\mu$g aliquot of total cellular RNA was reverse transcribed using random hexanucleotide as primer. A complementary DNA (cDNA) equivalent of 0.5 ng of RNA was amplified in a 50 $\mu$l reaction mix during 35 cycles (1 min denaturation at 94°C, 1 min annealing at 60°C, 1 min extension at 72°C). Each experiment included a positive control [cDNA of IL-2-stimulated lymphokine activated killer (LAK) cells] and a negative control consisting either of reaction mix without cDNA or of sample RNA that had not been reverse transcribed. For comparison of IL-10 mRNA levels in different tissue specimens, cDNAs were first adjusted to equal concentrations of $\beta$-actin by competitive PCR as recently described (Überla *et al.*, 1991). Briefly, serial 10-fold and subsequently 2-fold dilutions of cDNA were amplified in the presence of a fixed amount of $\beta$-actin control fragment, in order to determine exactly the amount of cDNA required to achieve equal band intensities for both fragments. The so-equalised cDNAs were then analysed for IL-10 mRNA content. Specificity of amplification products was verified by restriction analysis with two enzymes indicative for the expected amplified sequence (data not shown). Electrophoresis of 20 µl of PCR reaction or digestion product on 1.5% agarose gel containing ethidium bromide was performed to evaluate amplification and size of the generated fragments in comparison with the KB DNA ladder (Gibco/BRL, Gaithersburg, MD, USA). Primer sequences for β-actin (positions 103–122 and 642–619) and IL-10 (positions 283–309 and 608–634) were taken from Yamamura *et al.* (1991) and crossed intron–exon boundaries in genomic DNA.

*Assays for IL-10 reactivity*

Melanoma cells and melanocytes were incubated for 48 h at $1 \times 10^6$ cells ml$^{-1}$ and supernatants (SNs) were collected. SNs were assayed for IL-10 activity by an IL-10 enzyme-linked immunosorbent assay (ELISA) kit according to the supplier's recommendations (Cytoscreen, BioSource International, Camarillo, CA, USA). The lower detection limit of the ELISA was 18 pg ml$^{-1}$ for human IL-10. In addition, IL-10 activity was tested in the murine D36 mast cell proliferation assay (Schlaak *et al.*, 1993). Functionally, human IL-10 has been shown to possess the same biological activity as murine IL-10 when tested on murine cell targets (Vieira *et al.*, 1991). Briefly, aliquots of the SNs and serial 2-fold dilutions were added to microtitre plates containing $1 \times 10^3$ D36 cells per well and murine IL-4 (8 units ml$^{-1}$) in a final volume of 200 µl. Cultures were incubated for 24 h at 37°C, pulse labelled for another 18 h with 0.1 µCi per well [$^3$H]thymidine (5 Ci mmol$^{-1}$ specific activity) and harvested on glass fibre filters for measurement of [$^3$H]thymidine uptake in a scintillation counter. One unit per ml of IL-10 activity was defined as the reciprocal of SN dilution required for half-maximal proliferation. In order to confirm specificity of bioactivity, D36 cell proliferation was determined in the presence of the neutralising rat anti-human IL-10 monoclonal antibody JES3-19F1 (IgG 2a) (2.5 µg ml$^{-1}$) (De Waal Malefyt *et al.*, 1991*b*) and an isotype-matched control antibody (2.5 µg ml$^{-1}$). JES3-19F1, in concentrations up to 0.1 µg ml$^{-1}$, completely blocked biological activity of 5 units ml$^{-1}$ recombinant human IL-10.

### Results

*RT–PCR analysis of IL-10 mRNA expression in tissue specimens*

To investigate the expression of IL-10 mRNA in small specimens of human primary melanoma and melanoma metastases, we extracted total cellular mRNA from tissue specimens, and reverse transcribed it to cDNA. To provide meaningful comparison between different tissue samples, cDNAs were normalised to β-actin PCR product by competitive amplification of the cDNAs with a β-actin control fragment (see Figure 1). These standardised cDNA samples were analysed for IL-10 transcripts using IL-10-specific primers. Four out of five primary melanomas expressed IL-10 mRNA, whereas four out of five matched normal skin samples showed no IL-10 transcripts at all, and one skin sample barely expressed IL-10 mRNA (Figure 1). In addition, all three melanoma metastases analysed clearly expressed IL-10 mRNA.

*Expression of IL-10 mRNA and secretion of IL-10 protein in cell cultures*

To determine whether melanoma cells themselves express IL-10 mRNA and may be a source of IL-10 mRNA observed in melanoma tissues, we screened 13 human melanoma cell lines for gene expression of IL-10 by RT–PCR analysis as compared with normal melanocytes, keratinocytes and fibroblasts. As illustrated in Figure 2a, 3 out of 13 melanoma cell lines strongly expressed mRNA of IL-10, one showed moderate and three weak expression, whereas six cell lines were negative. In melanocyte cultures from seven different donors, weak but clearly detectable levels of IL-10 mRNA were consistently observed (Figure 2b), while fibroblasts and keratinocytes (cultures from two different donors) did not express IL-10 mRNA (data not shown). The amounts of cDNA analysed were similar in various samples and also between different cell types, as could be shown by amplification of β-actin mRNA (Figure 2). We next analysed SNs of these 13 melanoma cell lines and seven melanocyte cultures for IL-10 reactivity. The level of IL-10 mRNA detected in melanoma cell lines by RT–PCR correlated with the amount of IL-10 found in SNs, ranging form 0.57 ng ml$^{-1}$ to 3.40 ng ml$^{-1}$ IL-10 (Table I). Only cell line A375 which had shown moderate expression of IL-10 mRNA did not secrete measurable levels of IL-10 protein, nor did the other three cell lines with weak IL-10 transcripts. SNs of all seven melanocyte cultures tested did not contain measurable IL-10 protein. To confirm biological activity of melanoma cell-

**Figure 1** Expression of IL-10 mRNA in primary malignant melanoma (PM), melanoma metastases (MM) and normal skin (NS). The cDNAs derived from lesions were normalized to β-actin PCR product by competitive amplification with a β-actin control fragment (c.f.) and then analysed for IL-10 transcripts (see arrow) using IL-10-specific oligonucleotide primers.

**Figure 2** Expression of IL-10 mRNA (see arrow) in human melanoma cell lines (**a**) (lanes 2–16: Bro, A375, NKI-4, SKMel-28, MeWo, IGR-39, Mel-57, O-Mel-II, Mel-2a, SKMel-13, SKMel-19, IGR-37, M5, LAK, negative control) and melanocyte cultures (**b**) (lanes 2–7: StNM-1, StNM-7, StNM-8, Lei-1, Mel-4, Mel-5, Mel-6, LAK, negative control). Lane 1 in each case shows the KB DNA ladder. The amount of cDNA analysed was similar in different samples and cell types as shown by PCR amplification of β-actin mRNA.

**Table I** Expression of IL-10 reactivity in human melanoma cell lines

| *Melanoma cell line* | *ELISA* (ng ml$^{-1}$) | *Bioassay* (units ml$^{-1}$) |
|---|---|---|
| A375 | 0 | 0 |
| NKI-4 | 2.070 | 3 |
| IGR-39 | 0.570 | 1,5 |
| SKMel-19 | 3.400 | 6 |

IL-10 activity secreted in culture media per $10^6$ cells ml$^{-1}$ and 48 h, as determined by (a) an IL-10-specific ELISA and by (b) the D36 mast cell proliferation assay. Results are given as average of three experiments; s.d. values were less than 10%.

derived IL-10, culture SNs were tested in the D36 assay. SNs of the three melanoma lines that had been found positive in the IL-10 ELISA contained activities which varied between 1 and 8 units $ml^{-1}$, and could be specifically neutralised by an anti-IL-10 monoclonal antibody.

### Discussion

The results presented here demonstrate that mRNA for IL-10 is found in tissues of primary tumours and metastases but not in adjacent normal skin of patients with malignant melanoma. The PCR technique used does not enable us to distinguish which cells in the tumour tissue are producing IL-10 mRNA: it may originate either from infiltrating cells known to be able to produce IL-10 in humans, e.g. B cells, T cells and monocytes (Vieira *et al.*, 1991; De Waal Malefyt *et al.*, 1991*a*; Yssel *et al.*, 1992; Del Prete *et al.*, 1993) and/or from melanoma cells themselves. The mere presence of lymphocytic infiltrates in skin lesions does not necessarily signify IL-10 expression, as has been shown for epithelial tumours (Yamamura *et al.*, 1993). The present finding, that metastatic lesions which normally show less inflammatory response than primary melanomas (Payan *et al.*, 1970) expressed levels of IL-10 mRNA similar to the primary tumours, together with the fact that several melanoma cell lines constitutively secreted IL-10 rather argues for the possibility that IL-10 in melanoma tissue may be produced by the tumour cells themselves. However, to characterise unequivocally IL-10-expressing cells in the melanoma tissues either *in situ* hybridisation or immunohistochemistry has to be applied in further investigations. Melanocytes, the benign counterparts of melanoma cells, weakly expressed IL-10 mRNA but did not produce detectable IL-10 protein, nor did fibroblasts and keratinocytes. In one out of five normal skin samples examined in the present study, IL-10 mRNA was detected. In support of these results, constitutive expression of IL-10 mRNA has been found occasionally in unstimulated murine skin and reproducibly in hapten-stimulated skin samples (Enk & Katz, 1992). Accordingly, hapten-stimulated murine keratinocytes expressed IL-10 mRNA and protein. Possibly, appropriate stimulation may lead to IL-10 expression by human keratinocytes as well.

Interestingly, selective expresssion of IL-10 mRNA has also been found in ovarian tumours but not in normal ovaries and ovarian tumour cell lines (Pisa *et al.*, 1992). Moreover, our results are in line with recent published PCR data on the comparison of cytokine profiles in another malignant tumour of the skin, namely basal cell carcinoma, to that of seborrhoeic keratosis, a benign hyperplasia of epidermis (Yamamura *et al.*, 1993). The authors noted prominent mRNA expression of IL-4 and IL-10 in carcinoma specimens, but of IL-2 and interferon gamma in irritated seborrhoeic keratosis. Most recently, the constitutive production of IL-10 mRNA and protein by several human carcinoma cell lines has been reported (Gastl *et al.*, 1993). IL-10 was produced predominantly by epithelial cancer cell lines such as colon carcinoma, malignant melanoma and renal cell carcinoma. While these studies were restricted to established cell lines we could additionally demonstrate expression of IL-10 in tissues of primary and metastatic melanomas. IL-10 is a pleiotropic cytokine, and there is accumulating evidence from *in vitro* systems that IL-10 may play an important role not only in the regulation of T cell responses, but also as an anti-inflammatory mediator *in vivo* (Richter *et al.*, 1993). It is therefore intriguing to speculate that IL-10 suppresses anti-tumour immune responses and thereby facilitates tumour growth and development. IL-10 in human tumours could act, for example, by reduction of cell-mediated cytotoxicity and antigen presentation by monocytes (De Waal Malefyt *et al.*, 1991*b*), or by down-regulation of tumour necrosis factor α and/or interferon gamma production, two tumoricidal products of monocytes and T cells (De Waal Malefyt *et al.*, 1991*a*; Ralph *et al.*, 1992; Tosato & Taga, 1992; Del Prete *et al.*, 1993).

Most recently, production of IL-10 mRNA and IL-10 protein could be detected *in situ* in AIDS lymphomas (Emilie *et al.*, 1992). Expression of IL-10 was associated with the presence of Epstein–Barr virus in lymphomatous cells. Human IL-10 exhibits extensive sequence homology to a previously uncharacterised open reading frame in the Epstein–Barr virus genome, BCRF-1 (Vieira *et al.*, 1991). The protein product of BCRF-1, designated viral IL-10, shares most properties with IL-10, including cytokine synthesis-inhibtory activity on T cells (Hsu *et al.*, 1990) and suppression of antigen-specific proliferative T-cell response as a result of down-regulation of class II major histocompatibility complex molecules on monocytes (De Waal Malefyt *et al.*, 1991*b*). The possibility that messages detected in the PCR analysis of melanoma lesions may result from BCRF-1 expression is ruled out, since PCR primers were designed to specifically amplify mRNA of human IL-10 but not mRNA of viral IL-10 (Hsu *et al.*, 1990; Vieira *et al.*, 1991).

Our present data demonstrate the selective expression of IL-10 mRNA in human melanoma tissue. In addition, production of biologically active IL-10 has been found in some melanoma cell lines. There are several reports that melanoma cell lines express mRNAs and also proteins for various cytokines, e.g. IL-1, IL-6, IL-8, IL-10, tumour necrosis factor α and granulocyte colony-stimulating factor (Colombo *et al.*, 1992, and reviewed therein; Gastl *et al.*, 1993). These and other cytokines are likely to be involved in the immune response to cancer. At this time it is unclear what the net effects of multiple cytokines are on the outcome of the host response to tumour. For unknown reasons some (about 20%) but not all melanoma patients respond to IL-2 immunotherapy (Rosenberg, 1991). The presence of IL-10 in melanoma lesions, whatever its source may be, might influence the immune response to this tumour. The role of IL-10 as a possible prognostic marker for successful immunotherapy remains to be investigated.

We would like to thank M.-V. Odenwald for excellent technical assistance and R. Coffman (DNAX, Palo Alto, CA, USA) for providing us with the monoclonal antibody JES3-19F1.

### References

COLOMBO, M.P., MACCALLI, C., MATTEI, S., MELANI, C., RADRIZZANI, M. & PARMIANI, G. (1992). Expression of cytokine genes, including IL-6, in human malignant melanoma cell lines. *Melanoma Res.*, **2**, 181–189.

DE WAAL MALEFYT, R., ABRAMS, J., BENNETT, B., FIGDOR, C.G. & DE VRIES, J.E. (1991*a*). Interleukin 10 (IL-10) inhibits cytokine synthesis by human monocytes: an autoregulatory role of IL-10 produced by monocytes. *J. Exp. Med.*, **174**, 1209–1220.

DE WAAL MALEFYT, R., HAANEN, J., SPITS, H., RONCAROLO, M.-G., TE VELDE, A., FIGDOR, C., JOHNSON, K., KASTELEIN, R., YSSEL, H. & DE VRIES, J.E. (1991*b*). Interleukin 10 (IL-10) and viral IL-10 strongly reduce antigen-specific human T-cell proliferation by diminishing the antigen-presenting capacity of monocytes via downregulation of class II major histocompatibility complex expression. *J. Exp. Med.*, **174**, 915–924.

DEL PRETE, G., DE CARLI, M., ALMERIGOGNA, F., GIUGIZI, M.G., BIAGIOTTI, R. & ROMAGNANI, S. (1993). Human IL-10 is produced by both type 1 helper (Th1) and type 2 helper (Th2) T cell clones and inhibits their antigen-specific proliferation and cytokine production. *J. Immunol.*, **150**, 353–360.

EBERLE, J., KRASAGAKIS, K., GARBE, C. & ORFANOS, C.E. (1993). Proliferation and morphology of melanoma cells and benign melanocytes under varying culture conditions. *Melanoma Res.*, **3**, 107–112.

EMILIE, D., TOUITOU, R., RAPHAEL, M., PEUCHMAUR, M., DEVERGNEE, O., REA, D., COUMBRARAS, J., CREVON, M.-C., EDELMAN, L., JOAB, I. & GALANAUD, P. (1992). In vivo production of interleukin-10 by malignant cells in AIDS lymphomas. *Eur. J. Immunol.*, **22**, 2937–2942.

ENK, A.H. & KATZ, S.I. (1992). Identification and induction of keratinocyte-derived IL-10. *J. Immunol.*, **149**, 92–95.

GASTL, G.A., ABRAMS, J.S., NANUS, D.M., OOSTERKAMP, R., SILVER, J., LIU, F., CHEN, M., ALBINO, A.P. & BANDER, N.H. (1993). Interleukin-10 production by human carcinoma cell lines and its relationship to interleukin-6 expression. *Int. J. Cancer*, **55**, 96–101. .

HOCK, H., DORSCH, M., KUNZENDORF, U., QIN, Z., DIAMANTSTEIN, T. & BLANKENSTEIN, T. (1993). Mechanisms of rejection induced by tumor cell-targeted gene transfer of interleukin 2, interleukin 4, interleukin 7, tumor necrosis factor, or interferon gamma. *Proc. Natl Acad. Sci. USA*, **90**, 2774–2778.

HSU, D.-H., DE WAAL MALEFYT, R., FIORENTINO, D.F., DANG, M.-N., VIEIRA, P., DE VRIES, J.E., SPITS, H., MOSMANN, T.R. & MOORE, K.W. (1990). Expression of interleukin-10 activity by Epstein–Barr virus protein BCRF1. *Science*, **250**, 830–832.

PAYAN, H.M., GILBERT, E.F. & JABOBS, W.H. (1970). Lymphocytic reaction around primary and metastatic melanomas. *South. Med. J.*, **63**, 1350–1354.

PISA, P., HALAPI, E., PISA, E.K., GERDIN, E., HISING, C., BUCHT, A., GERDIN, B. & KIESSLING, R. (1992). Selective expression of interleukin 10, interferon-γ, and granulocyte-macrophage colony-stimulating factor in ovarian cancer biopsies. *Prôc. Natl Acad. Sci. USA*, **89**, 7708–7712.

RALPH, P., NAKOINZ, I., SAMPSON-JOHANNES, A., FONG, S., LOWE, D., MIN, H.-Y. & LIN, L. (1992). IL-10, T-lymphocyte inhibitor of human blood cell production of IL-1 and tumor necrosis factor. *J. Immunol.*, **148**, 808–814.

RICHTER, G., KRÜGER-KRASAGAKES, S., HEIN, G., HÜLS, C., SCHMITT, E., DIAMANTSTEIN, T. & BLANKENSTEIN, T. (1993). Interleukin 10 transfected into Chinese hamster ovary cells prevents tumor growth and macrophage infiltration. *Cancer Res.*, **53**, 4134–4137.

ROSENBERG, S.A. (1991). Immunotherapy and gene therapy of cancer. *Cancer Res.*, **51** (Suppl.), 5074–5079.

RUSSEL, S.J. (1990). Lymphokine gene therapy for cancer. *Immunol. Today*, **11**, 196–200.

SCHLAAK, J.F., SCHMITT, E., HÜLS, C., MAYER ZUM BÜSCHFELDE, K.-H. & FLEISCHER, B. (1994). A sensitive and specific bio-assay for the detection of human interleukin 10. *J. Immunol. Methods*, **168**, 49–54.

TOPALIAN, S.L., SOLOMON, D. & ROSENBERG, S.A. (1989). Tumor-specific cytolysis by lymphocytes infiltrating human melanomas. *J. Immunol.*, **142**, 3714–3725.

TOSATO, G. & TAGA, K. (1992). IL-10 inhibits human T cell proliferation and IL-2 production. *J. Immunol.*, **148**, 1143–1148.

ÜBERLA, K., PLATZER, C., DIAMANTSTEIN, T. & BLANKENSTEIN, T. (1991). Generation of competitor DNA fragments for quantitative PCR. *PCR Method. Appl.*, **1**, 136–139.

VIEIRA, P., DE WAAL-MALEFYT, R., DANG, M.-N., JOHNSON, K.E., KASTELEIN, R., FIORENTINO, D.F., DE VRIES, J.E., RONCAROLO, M.-G., MOSMANN, T.R. & MOORE, K.W. (1991). Isolation and expression of human cytokine synthesis inhibitory factor cDNA clones: homology to Epstein–Barr virus open reading frame BCRF1. *Proc. Natl Acad. Sci. USA*, **88**, 1172–1176.

YAMAMURA, M., UYEMURA, K., DEANS, R.J., WEINBERG, K., REA, T.H., BLOOM, B.R. & MODLIN, R.L. (1991). Defining protective responses to pathogens: cytokine profiles in leprosy lesions. *Science*, **254**, 277–279.

YAMAMURA, M., MODLIN, R.L., OHMEN, J.D. & MOY, R.L. (1993). Local expression of anti-inflammatory cytokines in cancer. *J. Clin. Invest.*, **91**, 1005–1010.

YSSEL, H., DE WAAL MALEFYT, R., RONCAROLO, M.-G., ABRAMS, J.S., LAHESMAA, R., SPITS, H. & DE VRIES, J.E. (1992). IL-10 is produced by subsets of human CD4$^+$ T cell clones and peripheral blood T cells. *J. Immunol.*, **149**, 2378–2384.